# Epigenetics of maternal-fetal interface immune microenvironment and placental related pregnancy complications

**DOI:** 10.3389/fimmu.2025.1549839

**Published:** 2025-04-03

**Authors:** Xueqin Ma, Xin Chen, Xuefeng Mu, Min Cao, Yan Zhang

**Affiliations:** Department of Obstetrics and Gynecology, Renmin Hospital of Wuhan University, Wuhan, China

**Keywords:** epigenetics, DNA methylation, histone modification, non-coding RNA, RNA methylation, placental interface, immune microenvironment

## Abstract

Epigenetic regulation of placental development and pregnancy-related disease processes has recently been a hot research topic. Implantation and subsequent placental development depend on carefully orchestrated interactions between fetal and maternal tissues, involving a delicate balance of immune factors. Epigenetic regulation, which refers to altering gene expression and function without changing the DNA sequence, is an essential regulatory process in cell biology. Several epigenetic modifications are known, such as DNA methylation, histone modifications, non-coding RNA regulation, and RNA methylation. Recently, there has been increasing evidence that epigenetic modifications are critical for the immune microenvironment at the maternal-fetal interface. In this review, we highlight recent advances in the role of epigenetics in the immune microenvironment at the maternal-fetal interface and in epigenetic regulation and placenta-associated pregnancy complications.

## 1 Introduction

Pregnancy is a physiological state in which the maternal immune system smoothly tolerates hemizygous fetal tissues, and several immune mechanisms at the maternal-fetal interface act synergistically to protect the fetus from rejection (1). The percentage and function of immune cells *in utero* changes dynamically at different stages of pregnancy (2). During normal pregnancy, dynamic interactions between trophoblast cells and decidual immune cells are required to provide a suitable immune microenvironment for successful embryo implantation and normal fetal development (3). The most important immune cells in the human decidua include uterine natural killer (uNK) cells, different T-cell subpopulations, dendritic cells, and macrophages (4). In addition, humoral factors such as sex hormones and cytokines secreted by several immune and non-immune cells play an important role in immune tolerance and pregnancy maintenance (5). An in-depth understanding of the regulatory mechanisms of the immune microenvironment in fetal and maternal tissues will help to understand better the pathophysiological characteristics of pregnancy-related complications such as infertility, miscarriage, and pre-eclampsia (PE), and at the same time, provide a foundation for improving adverse perinatal outcomes (6).

Epigenetics is defined as protein expression changes that occur without alterations in the DNA sequence. It can be stably transmitted during cell proliferation and organismal development and is closely linked to various pathophysiological processes (7). Epigenetics is a cutting-edge discipline that has gradually developed while studying many life phenomena that do not correspond to traditional genetics. The regulatory modes of action of this process include but are not limited to, DNA methylation, histone modification, non-coding RNA (ncRNA) modification, and RNA methylation. These modes of regulation act individually or interact with each other (8, 9).

The pivotal role of epigenetic regulation in developing the maternal-fetal immune microenvironment has garnered significant scientific interest, with many recent studies published. This paper aims to provide a review of the epigenetic mechanisms of the immune microenvironment at the maternal-fetal interface and an overview of recent advances in epigenetic regulation and placenta-associated pregnancy complications.

## 2 An overview of epigenetic inheritance

**Figure 1:**
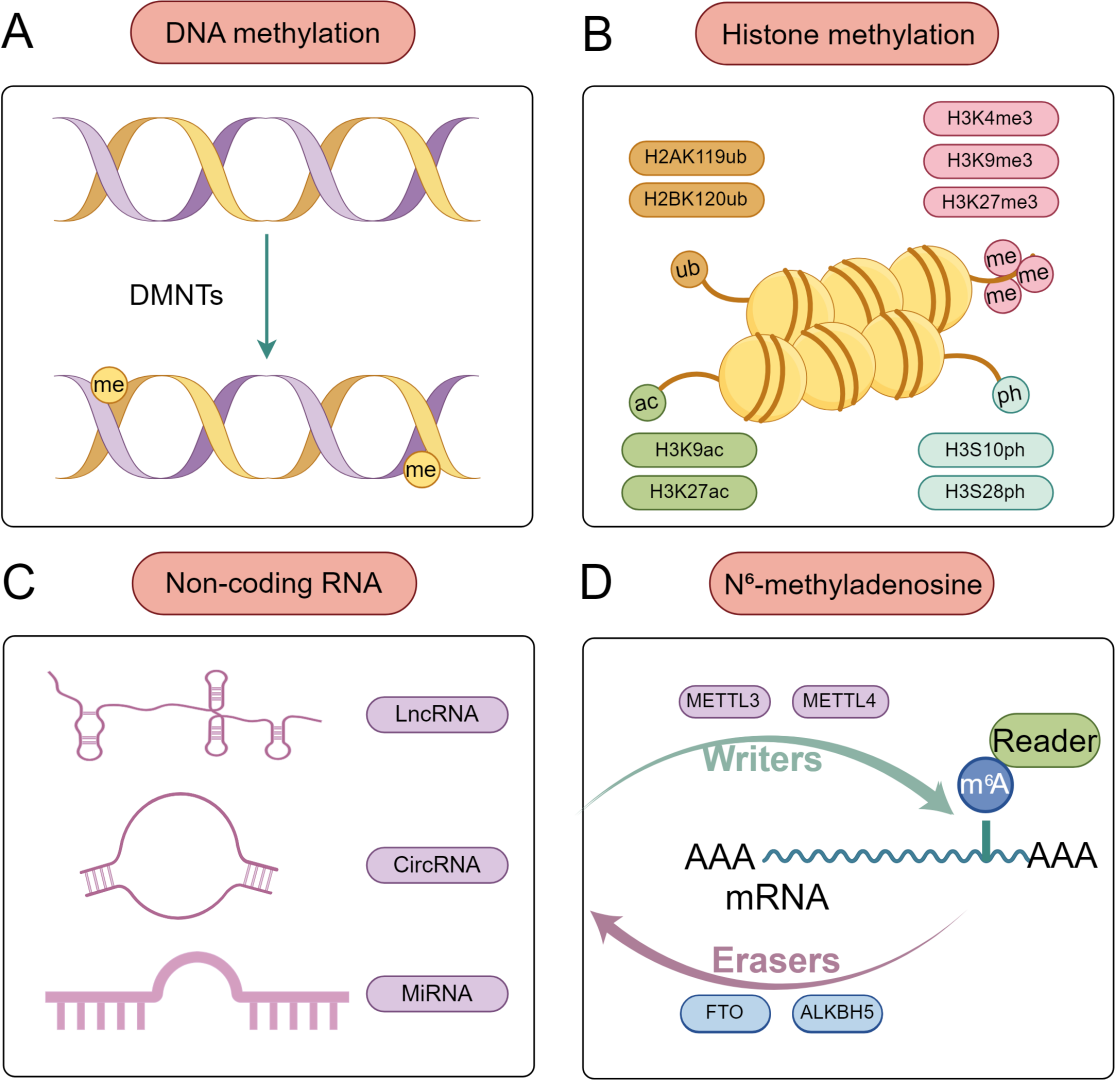
Common epigenetic modifications at the maternal-fetal interface. **(A)** DNA methylation. DNA methyltransferases (DNMTs) catalyze the covalent addition of a methyl group (-CH_3_) to the fifth carbon atom of cytosine **(C)** in DNA molecules, forming 5-methylcytosine (5mC). **(B)** Histone modifications. These include acetylation, phosphorylation, methylation, and ubiquitination, which play critical regulatory roles at the maternal-fetal interface by modulating chromatin structure and gene transcription activity. **(C)** Non-coding RNA. MiRNA mainly inhibits gene expression by binding to the 3 ‘untranslated region of mRNA; lncRNA is ncRNA with a length exceeding 200 nucleotides; CircRNAs are highly conserved circular ncRNAs that can act as miRNA sponges, bind to RNA binding proteins, or encode proteins. **(D)** N6-methyladenosine is the most common form of RNA methylation, which includes three regulatory systems: Writer, Eraser, and Readers. The Writer is responsible for catalyzing the addition of methyl groups to RNA, thereby promoting RNA methylation and ensuring its stability, such as METTL3 and METTL4. Eraser is responsible for mediating the demethylation modification process of RNA, such as FTO and ALKBH5. Readers can recognize and bind to the m6A site in mRNA.

“Epigenetics” describes altering gene expression and function without altering the DNA sequence, resulting in heritable phenotypes. The concept of epigenetic inheritance was first proposed by Waddington in the journal Endeavour in 1942 (10). The primary focus of genetic research is the inheritance or transmission of genotypes, whereas the process by which genotypes produce phenotypes is the domain of epigenetics. The process of epigenetic inheritance encompasses a range of mechanisms, including DNA methylation, histone modification, the regulation of ncRNA RNAs, and RNA methylation (**Figure 1**). These mechanisms influence the functions and properties of genes, primarily by regulating their transcription or translation processes (11).

### 2.1 DNA methylation

DNA methylation is prevalent in the human genome, and the normal methylation state of DNA is essential for the typical structure and function of the organism’s cells. DNA methylation, as a significant modality in the regulation of epigenetic mechanisms, refers to the process by which the 5th carbon atom on the CpG dinucleotide cytosine of a DNA sequence receives a methyl group from the methyl donor S-adenosyl methionine (SAM) using covalent bonding under the action of the enzyme DNA methyltransferase (DNMT). Methyl donor S-adenosyl methionine (SAM) is used to obtain a methyl group, and the product of this reaction is called 5-methylcytosine (5-mC) (12). DNA methylation is associated with gene silencing, where methylation of CpG sequences in the promoter or non-promoter regions can inhibit promoter binding to transcription factors or be recognized and bound by methyl CpG binding domain (MBD), thereby directly or indirectly inhibiting gene expression (13). DNA methylation occurs mainly at CpG islands in eukaryotes and is mainly regulated by DNMTs and methyl-binding proteins. Five DNMT proteins have been identified in mammals, but only DNMT1, DNMT3A, and DNMT3B have methyltransferase activity (14). DNMT1 can maintain the methylation status of hemimethylated DNA, while DNMT3A and DNMT3B preferentially act on unmethylated and hemimethylated DNA, and the TET protein (ten-eleven translocation, TET) can convert 5-methylcytosine to 5-hydroxymethylcytosine in order to induce DNA demethylation (15). At the maternal-fetal interface, DNA methylation exhibits significant differences in immune cells, which are crucial for maintaining pregnancy immune tolerance and placental development (16–18). However, abnormal DNA methylation regulation may affect embryonic development ability and interfere with the immune microenvironment of embryo attachment and maternal-fetal interface, leading to adverse pregnancy outcomes. Studies have shown that the DNA methylation pattern in human placenta has a reliable and significant correlation with preeclampsia (PE) and gestational diabetes (GDM) (19). Especially in PE patients, the DNA methylation status of non-imprinting genes in the placenta undergoes significant changes, with genes involved in cell adhesion, proliferation, invasion, and other functions showing particularly prominent methylation abnormalities (20).

### 2.2 Histone modifications

Histone modification refers to acetylation, phosphorylation, methylation, ubiquitination, and other modifications at histone amino acid sites (21). These modifications affect the compactness and accessibility of chromatin in different ways, affecting gene expression and, consequently, all aspects of biological physiology and developmental processes. At the same time, histone modification involves many modification sites and enzymes. Any abnormal link can lead to the occurrence of diabetes in pregnancy, recurrent abortion, PE, and other diseases.

#### 2.2.1 Histone acetylation

Acetylation of histones involves introducing acetyl groups to lysine residues in histone tails. This process is carried out by histone acetyltransferase (HAT), which adds the acetyl group to catalyze histone acetylation, and histone deacetylase (HDAC), which removes the acetyl group for deacetylation (22). Under normal conditions, histone acetylation is associated with transcription activation, whereas histone deacetylation is associated with gene silencing (23, 24). The level of acetylation is determined by the balance between HAT and HDAC activity and plays an essential role in chromatin remodeling and transcriptional regulation (25). Specifically, histone deacetylases 8 and 9 (HDAC8, HDAC9) are involved in regulating M1/M2 polarization of macrophages, while HDAC1 and HDAC2 can promote differentiation and fusion of placental trophoblast cells by regulating the development of trophoblast cells (26, 27).

#### 2.2.2 Histone methylation

Histone methylation is an important form of histone modification that involves adding one, two, or three methyl groups to certain amino acids in histones, known as mono-, bi-, and trimethylation, respectively. Although methylation can occur at many sites in histones, it mainly occurs at lysine (K) and arginine (R) residues in the tail (28–30). Here, we focus on histone lysine methylation because of its importance and range of vital functions. Histone lysine methylation mainly occurs on histones H3 and H4, and the six loci of H3K4, H3K9, H3K27, H3K36, H3K79, and H4K20 have been studied more (31, 32). Usually, H3K4, H3K36, and H3K39 are involved in transcriptional activation, while H3K9, H3K27, and H4K20 are involved in transcriptional repression or silencing, and thus the biological effects will vary according to different methylation sites and methylation levels (33). At the maternal-fetal interface, the process of sensitization of trophoblast cells is closely related to the enhancement of various histone modifications, including H3K4 trimethylation (H3K4me3), H3K9 acetylation (H3K9ac), and H3K27 acetylation (H3K27ac). The activation of natural killer cells (NK cells) is closely related to H3K4 monomethylation (H3K4me1) and H3K27 acetylation (H3K27ac) (33).

#### 2.2.3 Histone phosphorylation

Histone phosphorylation is achieved by adding phosphate groups to amino acid residues in histone tails (34). Several protein kinases catalyze this histone modification, are closely related to the cell cycle, and can affect the transcriptional activation of DNA by mediating the recruitment of DNA damage repair proteins (35). Almost all histones can be phosphorylated at specific sites, and together with other post-translational modifications, they regulate a variety of biological processes (36).

### 2.3 Non-coding RNA

The human genome is highly active at the transcriptional level, but only about 1.9% of the gene sequence is transcribed into proteins, while the rest is transcribed into ncRNAs (37). ncRNAs mainly consist of microRNAs (miRNAs), long-stranded non-coding RNAs (LncRNAs), and cyclic RNAs (circRNAs) (38). Among them, miRNAs are between 19 and 23 nucleotide units in length and mainly play a role in silencing gene expression by antisense inhibition of the 3’untranslated regions (3’UTR) of mRNAs (39); LncRNAs are a type of ncRNAs with a length of >200 nucleotide units (40); circRNAs are a type of highly conserved ncRNAs that form a closed-loop structure with covalent bonds, and their mechanism of action mainly includes acting as miRNA sponges, binding to RNA-binding proteins to regulate transcription, and encoding proteins (41). **Supplementary Table 1** summarizes the association of Maternal-fetal interface immune cells with ncRNAs.

### 2.4 RNA methylation

RNA methylation is the most common type of RNA modification, including N6 methyladenosine (m6A), 5-methylcytosine (m5C), N7 methylguanine (m7G), and N1 methyladenosine (m1A) (42). Among them, m6A is the most abundant RNA modification, mainly located in the 3 ‘untranslated region (3’ UTR) near the mRNA protein coding sequence and stops codons, regulating gene expression and biological functions by controlling RNA metabolism, alternative splicing, degradation, and translation processes (43, 44). The m6A methylation modification regulatory system mainly includes three categories: Writer, Eraser, and Readers. The writer mainly promotes RNA methylation and maintains its stability, with the most common molecules being METTL3 and METTL4. Eraser is responsible for mediating the demethylation modification process of RNA, such as molecular FTO and ALKBH5. Readers can recognize and bind to the m6A site in mRNA, shortening the half-life of mRNA. As an important post-transcriptional regulatory mechanism, RNA methylation can significantly affect the stability, intracellular localization, transport process, splicing mode, and translation efficiency of RNA (45). Research has shown that this epigenetic modification exhibits abnormal expression patterns in various reproductive system diseases, including pathological states such as PE, spontaneous abortion, endometriosis, and premature ovarian failure. The root cause of many pregnancy complications lies in poor implantation. Zhan Hong Zheng et al. (46) found that mice with Mettl3 deficiency were completely infertile due to implantation and decidualization failure, revealing that the m6A modification in the 5 ‘- UTR of Pgr mRNA mediated by METTL3 is closely related to the signal transduction of normal progesterone. In addition, the elevated level of RNA demethylase ALKBH5 in placental villus tissue of patients with recurrent spontaneous abortion (RSA) may be due to its ability to shorten the half-life of CYR61 mRNA in an m6A dependent manner and reduce the level of total m6A modification, affecting the invasiveness of the trophoblast and leading to miscarriage (47).

## 3 The role of epigenetic inheritance in the immune microenvironment at the maternal-fetal interface

**Figure 2:**
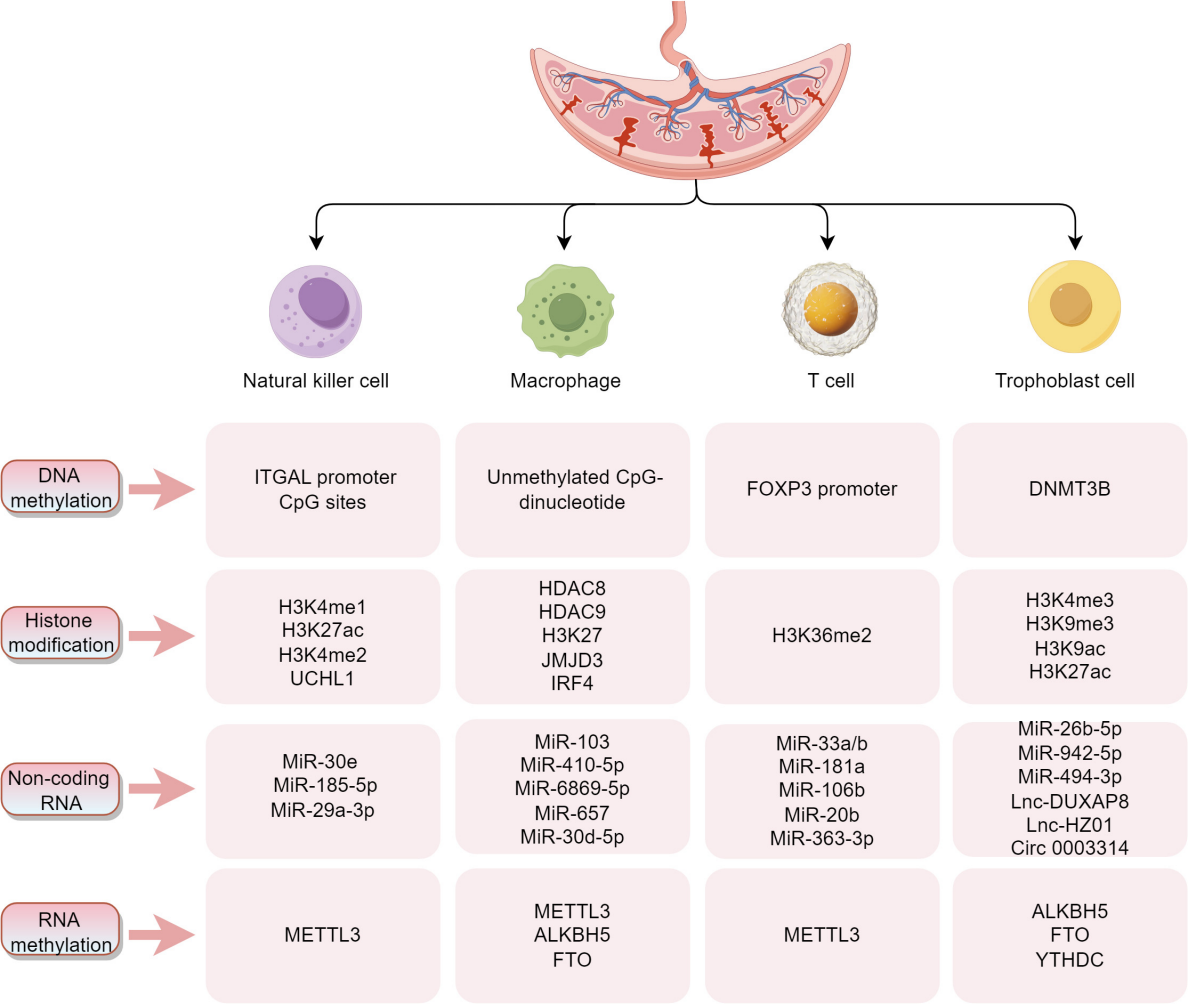
Summarizes the key molecules and modification sites associated with epigenetic regulation in four critical cell types (NK cells, macrophages, T cells, and trophoblasts) at the maternal-fetal interface. These epigenetic modifications, encompassing DNA methylation, histone modifications, non-coding RNAs (ncRNAs), and RNA methylation, play pivotal roles in regulating immune tolerance at the maternal-fetal interface and supporting placental development.

The maternal-fetal interface consists mainly of decidua from the mother and trophoblast cells from the embryo. The interactions between decidual immune cells and trophoblast cells form a vast network of cellular connections. The decidual immune system is constituted by different subpopulations of maternal immune cells, including decidual natural killer (dNK) cells, macrophages, and T cells, among others. Modern reproductive immunology suggests that imbalances in the immune microenvironment at the maternal-fetal interface may be involved in the development of pregnancy-related diseases. Increasing evidence suggests that epigenetic modifications are critical to the immune microenvironment at the maternal-fetal interface, and studies on the correlation between the dynamic function and compositional changes of vital immune cells (NK cells, macrophages, and T cells) and trophoblasts at the maternal-fetal interface and epigenetics are described in detail below (**Figure 2**).

### 3.1 The role of epigenetics in maternal-fetal interface NK cells

#### 3.1.1 Maternal-fetal interface NK cells

NK cells are intrinsic immune cells in the human body and are found in peripheral blood and endometrium (48). Among them, peripheral blood (pb) NK cells have two main subpopulations (CD56bright CD16dim and CD56dim CD16bright), and up to 90% of pNK cells are predominantly CD56dim CD16bright, and the primary function of pNK cells is highly cytotoxic (49). Compared to pbNK cells, dNK cells have a different phenotype; they resemble the CD56brightCD16dimpbNK subpopulation but have their unique characteristics, are mainly responsible for the production of cytokines and have poor cytotoxicity (50). Vento-Tormo et al. (51) have identified three significant subpopulations of dNK cells. In early pregnancy, NK cells are present in large numbers at the maternal-fetal interface, accounting for 70% to 80% of the decidual lymphocytes, whereas in the human decidua, 10% to 15% of the cells are decidual lymphocytes (52). Dnk cells are multifunctional, secreting various growth factors, angiogenic factors, chemokines, and cytokines. These not only synergistically promote the metamorphosis process of the endometrium and the remodeling of the spiral arteries of the uterus, thus laying a solid foundation for the growth of placental blood vessels and the construction of the placenta, but also exhibit a unique immune-regulatory mechanism. NK cells guide the migration of trophoblasts toward the fetal membranes and spiral arteries while preventing trophoblasts from being destroyed, thus regulating trophoblast invasion into the uterine wall and ensuring that the whole process is safe and smooth (52, 53).

#### 3.1.2 Maternal-fetal interface NK cells and DNA methylation

Decidual NK cells promote angiogenesis and trophoblast invasion and are closely associated with placental development (54). Epigenetic mechanisms such as DNA demethylation and histone acetylation have been shown to dynamically regulate gene transcriptional activity in immune cells, including CD8 (+) T cells and NK cells (18). However, research on the DNA methylation regulatory network in NK cells at the maternal-fetal interface is still limited. It is worth noting that the DNA methylation status is closely related to the activation level of NK cells: activated NK cells exhibit low methylation characteristics of CpG sites, while primitive NK cells maintain relatively high methylation levels, indicating that the methylation status of NK cells has significant plasticity and reproductivity (55). Hu et al. (17) found that DNA methylation in chorionic extravillous trophoblast (EVT) cells is regulated by dNK and the soluble molecules it secretes, which in turn affects EVT differentiation, adhesion, and migration. This discovery extends the function of dNK from classical immune regulation to epigenetic-mediated regulation of trophoblast development. In addition, Ana Sofia Cerdeira et al. (56) successfully induced dNK-like cell generation through pharmacological demethylation intervention, providing evidence for the core role of epigenetic mechanisms in dNK development. In summary, DNA methylation is not only a key epigenetic switch for the dynamic regulation of NK cell function but also affects the immune microenvironment and placental development at the maternal-fetal interface through dNK-mediated methylation remodeling. Future research needs to reveal the specific regulatory network of DNA methylation in dNK cells and its impact on pregnancy outcomes.

#### 3.1.3 Maternal-fetal interface NK cells and histone modification

Histone modifications are essential to promote normal NK cell development, and the histone H2A deubiquitinase MYSM1 regulates NK cells by controlling transcription factors, the lack of which severely impairs NK cell development (57). Giuseppe Sciumè et al. (58) found that activation of NK cells induced enhancer regions to H3K4me1 and H3K27ac histone modifications. Wiedemann et al. (59) used a multi-omics approach to analyze the complex interactions between cytokine signaling pathways in NK cells and found that IFN - α can enhance the epigenetic modification of the H3K4me3 promoter site. Ma et al. (60) demonstrated that SPA-VSMC has the potential to transform into CD56 dNK by identifying a small portion of dNK modified with H3K4me2 in the myosin heavy chain 11 (MYH11) promoter region through immunofluorescence-DNA *in situ* hybridization-neighbor-joining and chromatin immunoprecipitation experiments, which is the first demonstration that SPA-VSMC has the potential to transform into CD56 dNK. Zhang et al. (61) showed that ubiquitin C-terminal hydrolase L1 (UCHL1) can regulate decidualization through the JAK2/STAT3 signaling pathway. When UCHL1 is deficient, it can cause damage to decidualization during pregnancy in mice, leading to miscarriage, and is associated with a decrease in the number of decidual natural killer cells (dNKs). Through the above research, we can gain a deeper understanding of the epigenetic regulatory mechanisms of NK cells, as well as the pathogenesis of related diseases such as miscarriage.

#### 3.1.4 Maternal-fetal interface NK cells and non-coding RNAs

In recent years, multiple studies have revealed the important role of NK cells in pregnancy and their epigenetic regulatory mechanisms. Gamliel et al. (62) discovered a unique phenotype and transcriptional features of NKG2C high NK cell population in the decidua of women with multiple pregnancies. These cells showed increased accessibility at the Ifng and Vegfa loci and exhibited more vigorous IFN - γ and VEGF-A secretion. It provides important clues for understanding how microenvironmental changes during pregnancy induce epigenetic reprogramming of NK cells. In terms of miRNA regulation of NK cells, research (63) found through comparative analysis that 36 miRNAs were expressed explicitly in decidual NK cells. In comparison, two miRNAs were only expressed in peripheral blood NK cells, revealing molecular differences in NK cells from different sources. Another study (64) through miRNA profiling analysis of decidual NK cells from patients with unexplained recurrent spontaneous abortion (URSA), 50 differentially expressed miRNAs were identified, of which 49 were upregulated, and 1 was downregulated, indicating a close association between abnormal miRNA expression and URSA. Specifically, researchers have found through *in vitro* experiments that upregulated miR-30e can reduce the cytotoxicity of peripheral blood and decidual NK cells by targeting PRF1, inhibit Th1 tolerance phenotype, and induce Th2 immune dominance, thereby promoting the formation of a micro immune tolerance environment at the maternal-fetal interface (65). In addition, miR-185-5p is involved in the occurrence of RSA by interfering with VEGF expression and angiogenesis in decidual NK cells (66). Fang et al. (67) found that a decrease in miR-29a-3p levels in villous-derived exosomes of patients with URSA can increase IFN - γ levels in decidual NK cells. *In vivo*, experiments in mice revealed that vEXOs carrying miR-29a-3p can reduce embryonic resorption in RPL mice. However, despite significant progress in these studies, the epigenetic regulatory mechanisms of decidual NK cells have not been fully elucidated. Further in-depth research is needed regarding the proportion, functional status, and relationship with epigenetic regulatory mechanisms of decidual NK cells.

#### 3.1.5 Maternal-fetal interface NK cells and RNA methylation

In METTL3 deficient NK cells, the absence of m6A modification leads to a decrease in SHP-2 protein expression, inhibiting the activation of AKT and MAPK signaling pathways, and significantly reducing NK cell responsiveness to IL-15. In addition, the absence of METTL3 disrupts the homeostasis of NK cells. It inhibits their infiltration and function in the tumor microenvironment, ultimately accelerating the development of mouse tumors and shortening their survival period (68). Although the function of RNA methylation (especially m6A modification) in immune cells is gradually being revealed, research on its role in natural killer cells (NK cells) at the maternal-fetal interface is still relatively limited. Mother-fetal interface NK cells (NK cells) are crucial in early pregnancy embryo implantation, placental formation, and maternal-fetal immune tolerance. However, their functional regulatory mechanisms still need further exploration.

### 3.2 The role of epigenetics in maternal-fetal interface macrophages

#### 3.2.1 Maternal-fetal interface macrophages

Decidual macrophages are the second largest immune cell population (10%-20%) in the uterine decidua in early human pregnancy, second only to the percentage of dNK cells (60%-80%) (69). Notably, macrophages can be categorized into classically activated (M1) and alternatively activated (M2) phenotypes based on their function and the types of cytokines they produce (70). M1 macrophages are pro-inflammatory and are characterized by the production of higher levels of IL-12 and IL-23, as well as lower levels of IL-10, thus killing intracellular microbes and inducing Th1 immunity. In contrast, M2 macrophages produce higher levels of IL-10 and lower levels of IL-12 and IL-23 and perform functions such as anti-inflammatory and tissue remodeling, as well as the removal of apoptotic cells and debris (71). At the maternal-fetal interface, the number and ratio of M1/M2 macrophages change during different gestation periods. Specifically, M1-type macrophages predominate during the precomputation period, followed by a transition to a mixed population of M1 and M2 types; after the establishment of the placenta, M2-type macrophages dominate (72). Evidence suggests that an imbalance of M1/M2 macrophages leads to a pro-inflammatory microenvironment in the endometrium, which is not conducive to inducing fetal tolerance and further leads to pregnancy-related disorders such as recurrent miscarriages (73, 74). M1 and M2 macrophages play different roles at different gestational stages; thus, the maintenance of a normal pregnancy requires that the M1/M2 macrophage is in a state of equilibrium.

#### 3.2.2 Maternal-fetal interface macrophages and DNA methylation

A study (16) conducted genome-wide methylation analysis on maternal monocytes, fetal monocytes, decidual macrophages, and fetal placental macrophages (Hofbauer cells) using Illumina Infinium Human Methylation 27 BeadChip technology and found significant differences in DNA methylation among these cell populations. Notably, genes related to immune response are highly methylated in fetal cells, while maternal cells exhibit different methylation patterns. Meanwhile, excessive pro-inflammatory reactions at the maternal-fetal interface may jeopardize pregnancy maintenance. At the same time, DNA methylation may play a protective role in this process by regulating the anti-inflammatory function of macrophages. In premature birth studies, genome-wide placental DNA methylation analysis combined with methylated DNA immunoprecipitation sequencing (MeDIP-seq) technology revealed the gene functional characteristics of differentially methylated regions (DMRs), with a significant enrichment of Fc - γ receptor-mediated macrophage phagocytosis related pathways (75). This discovery suggests that DNA methylation may be involved in the mechanism of premature birth by regulating the phagocytic function of macrophages. In addition, unmethylated CpG dinucleotides derived from microorganisms can activate Toll-like receptor 9 (TLR-9), and these DNA activators exert phagocytic and clearance functions through the production of intrauterine immune cells (such as macrophages), thereby affecting the immune microenvironment at the maternal-fetal interface (76). These studies collectively reveal the important role of DNA methylation in regulating macrophage function at the maternal-fetal interface and maintaining pregnancy, providing a new perspective for a deeper understanding of the epigenetic mechanisms of pregnancy-related diseases.

#### 3.2.3 Maternal-fetal interface macrophages and histone modification

It was found that the expression of HDAC8 was reduced in decidual macrophages from patients with RSA, and the knockdown of HDAC8 inhibited M2 macrophage activation and promoted apoptosis of differentiated THP-1 (dTHP-1) macrophages through ERK pathway (26). Meng et al. (71) reported that nuclear factor-κ B ligand (RANKL) receptor activator, secreted by human embryonic trophoblasts and maternal decidual stromal cells, polarizes decidual macrophages to an M2 phenotype, which is mediated by activation of Akt/signal transducer and activator of transcription factor 6 (STAT6) signaling, and correlates with the up-regulation of the histone H3 lysine 27 demethylases, Jmjd3 and IRF4, in decidual macrophages. Liu et al. (77) found that Hdac9/HDAC9 deficiency promotes macrophage polarization toward M2 macrophages, while Hdac9/HDAC9 ablation significantly enhanced phagocytosis of fluorescent microspheres in M2 Raw 264.7 cells, but reduced the capacity of THP-1-derived M1 macrophages. These studies collectively reveal the key role of histone modifying enzymes in macrophage polarization and functional regulation.

#### 3.2.4 Maternal-fetal interface macrophages and non-coding RNAs

##### 3.2.4.1 miRNAs

NcRNA can regulate macrophage function and M1/M2 polarization, closely related to the occurrence and development of various pregnancy-related diseases. Some *in vitro* experimental studies have shown that specific miRNAs affect macrophage polarization by regulating key signaling pathways. For example, miR-103 and miR-410-5p inhibit the STAT1-mediated signaling pathway, preventing polarization of M1 macrophages and preventing RSA (78, 79). MiR-6869-5p induces M2 polarization in gestational diabetes by regulating PTPRO (80). On the contrary, miR-657 promotes gestational glucose by targeting FAM46C (81). In addition, miRNA-30d-5p derived from placental exosomes induces M2 polarization by inhibiting HDAC9 expression (82). The low-level miR-455-3p in decidual stromal cells inhibits the invasion of trophoblast cells by promoting macrophage polarization (83). MiR-146a-5p has been shown to increase embryo resorption rate and promote M2 polarization of macrophages in URSA (84). The article also briefly introduces the relationship between some miRNAs and macrophages (85).

##### 3.2.4.2 lncRNA

LncRNAs also participate in the regulatory network of macrophage polarization. Wu et al. (86) found that the expression of AOC4P was significantly up-regulated in trophoblast cells of patients with RSA. This molecule inhibits the degradation of EZH2 by regulating TRAF6, thus inhibiting glycolysis in trophoblast cells and actively participating in the polarization process of M2 macrophages. Xiujun Li et al. (87) confirmed through *in vitro* experiments that lncRNA MALAT1 not only regulates the proliferation and angiogenesis of mesenchymal stem cells (MSCs) but also promotes M2 polarization by regulating the expression of indoleamine 2,3-dioxygenase (IDO). LINC00240 could promote macrophage polarization toward the M2 type by regulating the miR-155/Nrf2 axis (88). The knockdown of LINC00221 negatively regulated the expression of miR-542-3p in trophoblasts to reduce macrophage migration and invasion (89). These studies provide a new perspective for elucidating the regulatory role of ncRNA in macrophage polarization. However, most current research is limited to *in vitro* experiments. The mechanism of action of lncRNA is highly complex, and its function may vary significantly depending on cell type and pathological state. Therefore, in the future, more *in vivo* experiments are needed to verify its biological functions and further explore its regulatory mechanisms and therapeutic potential in diseases by combining clinical samples.

#### 3.2.5 Maternal-fetal interface macrophages and RNA methylation

METTL3 participates in macrophage regulation through different mechanisms as a “writer” in RNA methylation. Low expression of METTL3 can inhibit the degradation of NOD1 and RIPK2 mRNA mediated by YTHDF1 and YTHDF2, thereby upregulating the NOD1 pathway and subsequently promoting LPS-induced macrophage inflammatory response (90). Another study showed that METTL3 could promote oxLDL-induced macrophage inflammatory response by activating STAT1 signaling (91). Zhao et al. (92) co-cultured ALKBH5 overexpressing cell lines with THP-1 and found that the N6 methyladenosine regulatory factor ALKBH5 damaged macrophage recruitment and M2 differentiation by reducing VEGF secretion in stromal cells, leading to miscarriage. Meanwhile, inhibiting VEGF signaling can help discover changes in macrophage polarization in different pregnancy complications (93). In addition, Cox analysis found that M2 macrophages were positively correlated with the m6A regulatory factor FTO and negatively correlated with CBLL1 (94).

### 3.3 The role of epigenetics in maternal-fetal interface T lymphocytes

#### 3.3.1 Maternal-fetal interface T lymphocytes

In early pregnancy, 30% to 45% of the decidual T cells are CD4+ T cells at the maternal-fetal interface, and 45% to 75% are CD8+ T cells (95). Under normal conditions, a dynamic balance is maintained between CD4+ and CD8+ T lymphocytes (96). CD4+ T cells are mainly helper T cells (Th) and regulatory T cells (Treg) (97). CD4+ helper T cells (Th) are classified into Th1, Th2, and Th17 cells according to the type of cytokines they secrete (98). Th1 cytokines promote macrophage activation and cytotoxicity and mainly mediate cellular immune responses (99). Th2 cytokines mediate humoral immune responses, promote eosinophil and mast cell differentiation, inhibit immune inflammation, reduce excessive damage, and have immunotoxic effects (100). After conception, maternal recognition of the fetus and maintenance of pregnancy is achieved through the immune balance between Th17/Treg, Th1/Th2 cells, and the dominant Th2-type cells (101). The large amount of Th2-type cytokines at the maternal-fetal interface inhibits the production of Th1-type cytokines through a negative feedback effect, suppresses TDTH and CTL cell activity, and thus inhibits rejection (102). Some subpopulations of maternal-fetal interface T cells help EVT invade the endometrium and promote embryo implantation and placenta formation, whereas other subpopulations are closely associated with pregnancy complications (103). The classical pattern of immune regulation in human pregnancy is a shift in maternal immune response from an inflammatory Th1 cytokine pattern to a Th2 pattern, with maternal-fetal interface decidual T cells playing a crucial role in regulating the placental microenvironment and recognizing fetal antigens (104).

#### 3.3.2 T-lymphocytes and DNA methylation at the maternal-fetal interface

Research has shown that the methylation levels of Th1/Th2 pathway genes exhibit dynamic changes at different stages of pregnancy. During normal pregnancy, the methylation levels of Th2 pathway genes change more significantly in the early stages of pregnancy. In contrast, the methylation levels of Th1 pathway CpG genes change more frequently in the late stages of pregnancy, suggesting that the temporal regulation of Th1/Th2 gene methylation is closely related to the maintenance of pregnancy (105). Abnormal DNA methylation can participate in the occurrence of pregnancy-related diseases through multiple pathways. It can disrupt the immune balance at the maternal-fetal interface by regulating the proliferation, differentiation, and cellular activity of Th and Treg cells, thereby promoting pathological processes such as recurrent RSA (106). Comparative analysis reveals that RSA patients exhibit abnormal FOXP3 promoter hypermethylation in decidual tissues, resulting in suppressed FOXP3 expression and subsequent disruption of maternal-fetal immune tolerance, potentially representing a key molecular pathway in RSA (107).

#### 3.3.3 T-lymphocytes and histone modification at the maternal-fetal interface

Histone methyltransferase Nsd2 was found to upregulate CXCR4 expression through H3K36me2 modification, promoting Treg cell recruitment into the decidua to ensure maternal-fetal immune tolerance (108).

#### 3.3.4 T-lymphocytes and non-coding RNA at the maternal-fetal interface

Schjenken et al. (109) found that miR-155 is required to expand regulatory T cells to mediate robust pregnancy tolerance in mice. Notably, the expression of miR-33a/b and miR-181a was significantly down-regulated in patients with RSA and led to a reduction in the number of Treg cells by negatively regulating the expression level of the vital molecule sphingosine-1-phosphate receptor-1 (S1PR1) (110). In patients with PE, miR-106b inhibits Treg differentiation by suppressing TNF - β expression, while miR-20b and miR-363-3p upregulate TH17 cell transcription factor ROR γ t/STAT3 and enhance TH17 activity, respectively, leading to Treg/TH17 imbalance (111). It is worth noting that environmental factors can also interfere with ncRNA networks. For example, Jamie L. McCall et al. (112) confirmed that prenatal cadmium exposure activates CD4+T cells in offspring by upregulating lncRNA Snhg7, suggesting that exogenous toxic substances may participate in the pathological process of pregnancy through epigenetic mechanisms.

#### 3.3.5 T-lymphocytes and RNA methylation at the maternal-fetal interface

METTL3 deficient CD4 (+) T cells disrupt the homeostasis and differentiation of immature T cells (113). By hybridizing Mettl3f/f mice with Foxp3Cre YFP mice and specifically knocking out Mettl3 in Tregs, the results showed that the offspring mice’s peripheral lymph nodes and spleen significantly increased and developed severe autoimmune diseases and infertility. Mechanism studies have shown that the absence of Mettl3/m6A leads to an increase in Socs mRNA levels, inhibiting the IL-2-STAT5 signaling pathway, which is crucial for the function and stability of Treg cells (114). In addition, METTL3-dependent m6A methylation plays a critical role in regulating follicular helper T cell (TFH) lineage differentiation. Research has found that the absence of METTL3 in CD4+T cells impairs the function of TFH cells, leading to a significant reduction in germinal center (GC) response in METTL3 deficient mice after acute viral infection (115).

### 3.4 The role of epigenetics in trophoblasts at the maternal-fetal interface

#### 3.4.1 Trophoblast cells at the maternal-fetal interface

EVT are one of the main coordinators of immunity at the maternal-fetal interface (116), actively creating an environment for tolerogenic phenotypes by expressing a unique set of major histocompatibility complex (MHC) molecules (117).

#### 3.4.2 Trophoblasts and DNA methylation

DNA methylation in trophoblast cells continues to change dynamically after implantation and throughout pregnancy, with the differentiation of cytotrophoblasts into syngeneic trophoblasts and the acquisition of an invasive phenotype in EVT, both of which involve extensive DNA methylation changes (118). The study (119) identified ZBTB24 as an epigenetic regulator that modulates E-cadherin expression and, thus, cell viability, differentiation, and migration in trophoblasts by altering DNA methylation in the promoter region. In addition, dNK can regulate trophoblast function by altering gene expression through DNA methylation (120). In summary, these findings reveal the core regulatory role of DNA methylation in nourishing cells and pregnancy maintenance at the maternal-fetal interface.

#### 3.4.3 Trophoblast and histone modification

During the sensitization process of BeWo trophoblast cells, histone markers associated with active transcription (H3K4me3, H3K9ac, and H3K27ac) significantly increased, while the levels of inhibitory histone modifications (H3K9me3 and H3K27me3) remained unchanged (121). This process is consistent with increased mRNA levels of EP300 and P300/CBP-related factors (PCAF). The binding of RNA polymerase II to specific gene promoters is enhanced during the process of trophoblast differentiation, which is closely related to active histone markers. Meanwhile, the downregulation of pro-inflammatory transcription factors after differentiation of multinucleated syncytiotrophoblasts is associated with reduced enrichment of H3K27Ac and H3K9Ac promoters, as well as enhanced binding of H3K9me3 to histone deacetylase 1 (122). Histone deacetylases 1 and 2 regulate the development of extrachorionic trophoblast cells and promote the differentiation and fusion of human placental trophoblast cells (27). In addition, glycolytic metabolism is involved in regulating the function of trophoblast cells. Yu et al. (123) found that glycolysis exhibited high activity in human trophoblast stem cells and cytotrophoblast cells, whereas it was significantly reduced in syncytiotrophoblast cells. However, when supplemented with the glycolytic derivative acetyl coenzyme A, the fusion function of the syncytiotrophoblast was restored. Of particular note, acetylation of H3K9, H3K18, H3K27, and H4K16 was particularly sensitive to glycolysis during syncytiotrophoblast fusion with the metabolism of acetyl-coenzyme A. These modifications regulated the promoters of relevant genes involved in syncytiotrophoblast fusion and metabolism of syncytiotrophoblast cells. In terms of pathological mechanisms, an *in vitro* experiment found that knocking down ACLY disrupts histone acetylation and IL-10 secretion in nourishing cells, thereby inhibiting M2 polarization of macrophages. This process is involved in the pathogenesis of RSA (124). Sumi et al. (125) found that LPS treatment increased SIRT1 expression in mouse placental tissue and human trophoblast cells and inhibited NLRP3 inflammasome activation in trophoblast cells by reducing oxidative stress, thereby reducing lipopolysaccharide-induced IL-1 β levels. These studies collectively reveal the important role of histone modifications in the differentiation, metabolic regulation, and pathological processes of trophoblast cells.

#### 3.4.4 Trophoblasts and non-coding RNAs

MiRNAs are equally important in regulating trophoblast function, and their abnormal expression is closely related to pregnancy-related diseases. Liang et al. (126) systematically summarized miRNA regulation on trophoblast function and its related signaling pathways, providing a theoretical basis for understanding the mechanisms of pregnancy diseases. Meanwhile, lncRNA also participates in trophoblast dysfunction through various mechanisms. The long noncoding RNA DUXAP8 is significantly upregulated in the placental tissue of PE and can inhibit biological functions such as proliferation, migration, and invasion of trophoblast cells. The research team constructed a pregnant rat PE model to further validate its molecular mechanism by mediating DUXAP8 overexpression through adenovirus vectors. The experimental results showed that DUXAP8 activates the AKT/mTOR signaling pathway, inhibits endoplasmic reticulum autophagy, and promotes the occurrence and development of PE (127). *In vitro* experiments, Inc-HZ01 with m6A RNA methylation modification forms a positive feedback loop with MXD1, which plays a vital role in regulating abortive and BPDE-exposed trophoblast cell function regulation. This loop increases Inc-HZ01 levels by enhancing the RNA stability of MXD1, which inhibits trophoblast proliferation and induces miscarriage (128). Circ_0003314 inhibits trophoblast function and induces cell apoptosis through the miR-26b-5p/IL1RAP signaling axis (129). In the PE study, circ_0015382 impaired trophoblast function through the miR-942-5p/NDRG1 axis, and its expression level was significantly elevated in the placental tissue of PE patients (130). In addition, Ou et al. (131) found through Pearson correlation analysis of placental tissue from 25 PE patients that the expression level of circ_0111277 was significantly higher than that of normal pregnancy placenta. Mechanism studies have shown that circ_0111277 participates in the occurrence of PE by regulating the miR-494-3p/HTRA1/Notch-1 signaling pathway, inhibiting the invasion and migration ability of trophoblast cells. These research results indicate that ncRNA participates in the precise regulation of trophoblast function through a complex regulatory network, providing potential molecular targets for diagnosing and treating pregnancy-related diseases. However, the exact molecular pathways of these regulatory mechanisms still need to be elucidated through more in-depth *in vivo* experimental studies, including the construction of conditional gene knockout animal models.

#### 3.4.5 Trophoblasts and RNA methylation

*In vitro* experiments showed that knocking down m6A demethylase ALKBH5 can promote the invasion of HTR-8/SVneo in trophoblast cells by regulating the stability of CYR61 mRNA, while overexpression of ALKBH5 has the opposite effect (132). Meanwhile, Chuanmei Qin et al. (133) found that the expression of ALKBH5 in the villus tissue of RSA patients was elevated compared with the control group and confirmed through *in vitro* experiments that ALKBH5-overexpression-inhibited RSL3-induced cell death in the trophoblast by promoting the expression of iron death-related gene FTL. In addition, the expression of m6A demethylase FTO protein increased in URSA trophoblast cells, inhibiting m6A modification of MEG3 and weakening the stabilizing effect of YTHDC1 protein on MEG3. MEG3 inhibits the expression of the TGF - β 1 gene by binding to the EZH2 protein and TGF - β 1 gene promoter, thereby affecting the invasion and proliferation of trophoblast cells (134). These findings reveal the key role of RNA methylation in regulating the function of trophoblast cells and its potential mechanisms in miscarriage.

## 4 Epigenetic regulation and placenta-related pregnancy complications

As mentioned above, epigenetic regulation plays a moderately important role in the immune microenvironment at the maternal-fetal interface, and their absence or dysfunction may lead to complications during pregnancy (135). It has been suggested that normal and abnormal pregnancies lead to changes in cell populations, which may be facilitated by epigenetic modifications induced by different pregnancy-associated processes (136). The placenta, a uniquely evolved organ that develops externally to the embryo, undergoes rapid growth and fulfills diverse roles throughout pregnancy, primarily ensuring a stable and protective milieu for fetal development. Notably, in recent years, shifts in lifestyle have coincided with a steady increase in the incidence of placental-related pregnancy disorders, exerting profound consequences on both maternal and neonatal health (137). **Table 1** summarizes the association between epigenetic regulation and placental related pregnancy complications.

**Table 1:** Epigenetic regulation and placenta-associated pregnancy complications.

Epigenetic modifications	Molecules	Mechanisms	Diseases	Reference
DNA Methylation	DNMT3A Downgrade	Mediates the non-dependent induction of DNA methylation by TGFBR1	Early onset severe pre-eclampsia	(138)
	Placental growth factor and Fms-associated tyrosine kinase-1 hypomethylation	━━	Pre-eclampsia	(139)
	DNA methylation at the CPG site	━━	Low birth weight	(140)
	Placental DNA methylation sites associated with birth weight	━━	Perinatal cardiometabolic status of the mother, chronic disease in later life of the offspring	(140)
	CD3 methylation in pregnancy	━━	Psychiatric symptoms such as depression and anxiety	(142)
Histone modification	Deletion of Men1, a member of the histone H3K4 methyltransferase complex	Disruption of terminal differentiation of stromal cells	Embryo resorption and pregnancy failure	(145)
	H3K18la downgrade	Influence on endometrial tolerance	Abortion	(146)
	Knockout of KAT8	Adjustment of H4K16ac/CDX2 axis	Vulnerable to embryo implantation failure induced miscarriage	(148)
	KDM5CK upward	Regulate the expression of TGFβ2 and RAGE	Recurrent spontaneous abortion	(149)
	H3K4me3 and H3K9ac downgrades	Regulated by Gal-2 and PPARγ	Pre-eclampsia	(150, 151)
	Down-regulation of HDAC 2 expression and activity in monocytes/macrophages	━━	Gestational diabetes	(153)
	Up-regulation of miR-153-3p	Mediated inhibition of trophoblast function by the IDO/STAT3 pathway	Unexplained recurrent miscarriage	(152)
Non-coding RNA	Up-regulation of miR-185-5p	Reduced VEGF Expression and Angiogenesis in dNK Cells	Recurrent spontaneous abortion	(66)
	Up-regulation of miR-515-5P	Reducing HDAC2 levels harms trophoblast cell biological behavior	Recurrent spontaneous abortion	(153)
	Up-regulation of miR-23a	Inhibition of HDAC2 and activation of NF-κB impede trophoblast migration and invasion and promote apoptosis	Pre-eclampsia	(154)
	Down-regulation of miR-199a-5p	Reduction of VEGFA expression to inhibit trophoblast invasion	Pre-eclampsia	(155)
RNA methylation	Upregulation of METTL3	Increase the level of m6A RNA methylation and hnRNPC1/C2 expression in trophoblasts	Pre-eclampsia	(157)
	Upregulation of METTL14	Increase the level of m6A RNA methylation and FOXO3a expression and inhibite trophoblast proliferation and invasion	Pre-eclampsia	(159)
	Upregulation of RBM15	RBM15 suppresses hepatic insulin sensitivity of offspring of gestational diabetes mellitus mice via m6A-mediated regulation of CLDN4	Gestational diabetes Mellitus	(160)
	Down-regulation of m6A methylation level	ALKBH5 regulates CYR61 mRNA stability through m6A dependent mechanism and affects trophoblast function	Recurrent spontaneous abortion	(132)
	Down-regulation of METTL3	METTL3 mediated ZBTB4 m6A RNA methylation modification inhibits trophoblast invasion ability	Recurrent spontaneous abortion	(161)

### 4.1 DNA methylation

Abnormal changes in DNA methylation are closely related to maternal health and fetal development. There are extensive DNA methylation changes in early-onset PE, among which TGFBR1 induces disease occurrence through a DNMT3A downregulation mediated DNA methylation-independent pathway (138). Meanwhile, the low methylation status of placental growth factor and Fms-related tyrosine kinase-1 further affects placental function (139). In terms of fetal development, changes in CpG methylation associated with maternal PE, pre-pregnancy weight loss, and type 2 diabetes risk are significantly associated with low birth weight, suggesting that placental DNA methylation may serve as a bridge between maternal metabolic status and the risk of chronic disease in offspring (140). In addition, epigenetic changes are also associated with other pregnancy-related diseases. Researchers recruited three decidua samples from patients with RSA and normal controls. They systematically identified key genes regulated by DNA methylation in the decidua and blood of RSA patients through genome-wide bisulfite sequencing (GWBS) and transcriptome sequencing. They found that 23 genes exhibited significant methylation and expression differences between RSA patients and healthy controls, with hypomethylated differential methylation regions (DMR) and upregulated differential gene expression (DGE) co-enriched in the Rap1, GnRH, and Estrogen signaling pathways. Additionally, 32 genes from these three pathways showed significant differences in DMR between RSA patients and the control group. Hi-MethylSeq analysis further revealed that SGK1 in RSA patients’ blood and decidua samples exhibited a high methylation state. At the same time, SGK3 and CREB5 also showed significant changes in methylation levels in the decidua (141). T lymphocyte CD3 methylation may mediate psychiatric symptoms such as depression and anxiety during pregnancy (142). The above research reveals the extensive role of epigenetic modifications in pregnancy-related diseases, providing a new perspective for the mechanism research of related diseases.

### 4.2 Histone modifications

HDAC 2 expression and activity are down-regulated in monocytes/macrophages of patients with gestational diabetes, suggesting the role of histone deacetylation in metabolic disorders (143). The difference in placental chromatin activity is associated with fetal growth restriction, and H3K27 acetylation may be involved in regulating placental function (144). Men1, a member of the H3K4 methyltransferase complex, is crucial for endometrial decidual transformation. Research has shown that abnormal differentiation of uterine stromal cells in mice with Men1 deficiency can lead to embryo resorption and pregnancy failure (145). Intriguingly, novel histone acetylation modifications, including H3K18la, have been identified as critical regulators of embryo implantation, with reduced levels potentially contributing to pregnancy loss. Optimal lactate concentrations facilitate endometrial cell proliferation and apoptosis regulation by enhancing histone acetylation, creating a favorable environment for successful embryo implantation (146, 147). Qianying Yang et al. (147) detected relatively high levels of lactate in the fetal membranes and endometrial tissues of pregnant sheep and found that lactate can induce H3K18 acetylation and regulate the balance of redox homeostasis and apoptosis in the endometrium to ensure successful implantation RSA is closely related to the abnormal proliferation and differentiation of early trophoblast cells. Histone acetyltransferase KAT8 activates downstream gene CDX2 through H4K16ac, regulating trophoblast cell proliferation. Its absence increases the risk of embryo implantation failure. Clinical analysis shows that decreased expression of KAT8, CDX2, and H4K16ac is associated with RSA (148). Meanwhile, H3K4-specific demethylase KDM5C affects the function of trophoblast cells by regulating H3K4me3 demethylation at the promoter of TGF β 2 and RAGE genes. Overexpression of KDM5C can lead to decreased proliferation and invasion ability of trophoblast cells. *In vivo* experiments further confirmed that overexpression of KDM5C leads to a significant increase in mouse embryo absorption rate (149). In addition, the expression of H3K4me3 and H3K9ac in the placenta of patients with PE is reduced, and these modifications are regulated by substances such as Gal-2 and PPAR γ, suggesting the potential role of epigenetic modifications in gestational hypertension (150, 151). In conclusion, histone modification plays an important role in embryo implantation, placental development, and pregnancy maintenance by regulating gene expression and cell function. Its abnormal expression may lead to complications such as pregnancy diabetes, RSA, and PE.

### 4.3 Non-coding RNA

Abnormal expression of ncRNA can affect pregnancy outcomes by regulating the function of trophoblast cells. In RSA, miR-153-3p is highly expressed in extracellular vesicles derived from decidual macrophages, inhibiting the proliferation and migration of trophoblast cells through the IDO/STAT3 pathway (152). Meanwhile, miR-185-5p expression was elevated in the villi of RPL patients, and further *in vitro* experiments showed that miR-185-5p leads to pregnancy failure by reducing VEGF expression in dNK cells and decreasing angiogenesis (66). In PE, multiple *in vitro* experiments have found that miRNA participates in disease development by regulating trophoblast cell proliferation, migration, and apoptosis. For example, miR-515-5P inhibits the proliferation, migration, and invasion of HTR-8/SVneo in trophoblast cells by reducing HDAC2 levels (153). MiR-23a inhibits HDAC2 and activates NF-κB, hindering the migration of trophoblast cells and promoting apoptosis (154). MiR-199a-5p inhibits trophoblast cell invasion by suppressing VEGFA expression (155). In addition, Fu et al. (156) have outlined the role of ncRNAs in pregnancy-related complications, which will not be described in detail here. The results indicate that ncRNA significantly contributes to the pathogenesis of RSA and PE by modulating crucial molecular pathways.

### 4.4 RNA methylation

N6-methyladenosine (m6A) RNA modification is a pivotal regulatory mechanism in pregnancy-related disorders. In PE, *in vitro* studies has found the methylation level of m6A RNA in the placental trophoblast is significantly increased, and the methyltransferase METTL3 participates in the pathogenesis by regulating the expression of hnRNPC1/C2 (157). METTL14 upregulates FOXO3a expression through m6A dependent mechanism, inhibiting proliferation and invasion of trophoblast cells (158). At the same time, the m6A-modified circRNA in PE placenta generally increases, while the m6A modification of circPAPP2 is enhanced, but its expression is reduced. Its stability is regulated by IGF2BP3 (159). In gestational diabetes (GDM), the researchers found that the overall mRNA m6A methylation level in the fetal liver of the mouse was significantly increased by constructing a GDM mouse model, and *in vitro* experiments revealed that RBM15 mediated m6A modification affected the insulin sensitivity of offspring by regulating the expression of CLDN4, leading to metabolic syndrome (160). Compared with normal early pregnant women, the m6A methylation level in placental villus tissue of RM patients was significantly reduced, while ALKBH5 expression was not explicitly regulated. *In vitro* experiments have shown that knocking down ALKBH5 promotes trophoblast invasion, while overexpression inhibits invasion. It may be that ALKBH5 governs the stability of CYR61 mRNA through a dependent mechanism, thereby affecting the function of the trophoblast (132). In addition, *In vitro* studies have found that reduced m6A modification mediated by METTL3 can increase ZBTB4 expression, impair the invasive ability of the trophoblast layer, and lead to adverse pregnancy outcomes (161). Suqi Wu et al. (162) further elaborated on the role of m6A modification in maternal-fetal crosstalk and its potential mechanisms in pregnancy-related diseases. These findings suggest that m6A modification plays an important role in pregnancy-related diseases by regulating different target genes. However, the complex regulatory network of m6A modification and its specific functions under different pathological conditions still needs further exploration. Future research should combine multi-omics analysis and *in vivo* experiments to deeply reveal the dynamic regulatory mechanism of m6A modification at the maternal-fetal interface.

## 5 Conclusion and prospect

Successful pregnancy depends on the dynamic balance of multiple immune factors in the maternal-fetal interface immune microenvironment (3). The production of maternal autoimmune antibodies and the imbalance of immune regulation at the maternal-fetal interface are important factors affecting embryo implantation and leading to RSA. In recent years, epigenetic research has revealed the key roles of DNA methylation, histone modification, ncRNA and RNA methylation in immune regulation at the maternal-fetal interface. Especially DNA methylation, as the most promising and sensitive biological marker in epigenetic modifications, its abnormalities are not only closely related to the occurrence of various immune-related diseases but can also affect the immune tolerance at the maternal-fetal interface, leading to the maternal immune system producing a defensive rejection response to the embryo (13, 54). Research on DNA methylation in oncology has shown that it occurs earlier than gene mutations and can directly obtain relevant genetic material from tissues, providing the possibility for early prevention and treatment of diseases. The pathogenesis of immune-type RSA is similar to that of tumors, characterized by immune cell dysfunction and immune response suppression. With the deepening of epigenetic research, DNA methylation is expected to become a key breakthrough point for targeted diagnosis and precise treatment of immune type RSA, promoting the rapid development of reproductive medicine.

The epigenetic study of immune regulation at the maternal-fetal interface provides a new perspective for analyzing pregnancy’s physiological and pathological mechanisms. By revealing the role of epigenetic modifications in the maternal-fetal interface immune microenvironment, researchers can gain a deeper understanding of the pathogenesis of pregnancy-related diseases and identify effective targets for immunotherapy. For example, dynamic changes in DNA methylation and histone modification may become key nodes in regulating maternal-fetal interface immune tolerance. In addition, the role of ncRN (such as miRNA and lncRNA) in regulating immune cell function at the maternal-fetal interface is gradually revealed. These findings lay a theoretical foundation for developing intervention strategies for pregnancy-related diseases based on epigenetics.

The fundamental causes of many placental diseases are still unclear. However, it is now widely believed that immune dysregulation at the placental interface can lead to many diseases (135, 163). Meanwhile, compared to genetic changes, epigenetic abnormalities are more straightforward to reverse. Drugs targeting specific epigenetic mechanisms involved in gene expression regulation, and even some nutrients, may become emerging tools for disease prevention or treatment. In this review, some evidence suggests that epigenetic modifications of inhibitory compounds or regulation of noncoding RNA expression through genetic tools may alleviate placental pregnancy-related diseases caused by immune dysregulation at the placental interface. These will provide new ideas for precise prevention and targeted treatment of diseases related to placental pregnancy.

Although significant progress has been made in the study of epigenetics in the maternal-fetal interface immune microenvironment, some limitations remain. Existing research has focused chiefly on single epigenetic modifications (such as DNA methylation or specific noncoding RNA). In contrast, research on the interactions and synergistic regulatory mechanisms between multiple epigenetic modifications is still insufficient. In addition, most studies rely on *in vitro* cell experiments or animal models, which make it difficult to fully simulate the complex immune microenvironment of the human maternal-fetal interface. However, a great deal of further research is still needed. Examples include (1) exploring how dysregulation of immunoregulation at the placental interface leads to epigenetic alterations in specific genes; (2) identifying human susceptibility genes in epigenetic alterations induced by dysregulation of immunoregulation at the placental interface; (3) demonstrating whether epigenetic alterations can be used as a biomarker for the early detection of, and therapeutic targeting for, pregnancy-associated diseases induced by dysregulation of immunoregulation at the placental interface; (4) developing novel inhibitors targeting epigenetic modifications;and (5) In-depth study of the interaction network and synergistic regulatory mechanism among various epigenetic modifications.
