# Supplementary material for: Epigenetics of maternal-fetal interface immune microenvironment and placental related pregnancy complications
Source: Front Immunol. 2025 Apr 3;16:1549839. doi: 10.3389/fimmu.2025.1549839 (PMC12003353; doi:10.3389/fimmu.2025.1549839)
Supplement: Supplementary file 1 [file Table1.docx]

Table 1. Maternal-fetal interface immune cells and non-coding RNAs

| Cell | Non-coding RNAs | Function/mechanism | Reference |
| --- | --- | --- | --- |
| NK cell | miR-30e | Targeting PRF1 reduces NK cytotoxicity and inhibits TH1 phenotypic tolerance inducing TH1 immunodominance | [65] |
|  | miR-185-5p | Interference with VEGF expression and angiogenesis in dNK cells | [66] |
|  | miR-29a-3p | Negative regulation of IFN-γ levels in dNK cells | [67] |
| Macrophage | miR-103  miR-410-5p | Inhibition of STAT1-mediated M1 macrophage polarisation | [78]  [79] |
|  | miR-6869-5p | Modulation of gestational diabetes PTPRO induced M2 polarisation | [80] |
|  | miR-657 | Targeting FAM46C in gestational diabetes promotes macrophage polarisation towards M1 | [81] |
|  | miR-30d-5p | Inhibition of HDAC9 expression induces macrophage polarisation to the M2 phenotype | [82] |
|  | miR-455-3p | Negative regulation of macrophage polarisation and inhibition of trophoblast invasive capacity | [83] |
|  | miR-146a-5p | Promoting polarisation of ecdysteroidal macrophages to the M2 phenotype | [84] |
|  | AOC4P | Regulation of TRAF6 inhibits EZH2 degradation and thus glycolysis in trophoblast cells; involved in M2 macrophage polarisation | [86] |
|  | LncRNA MALAT1 | Regulation of indoleamine 2,3-dioxygenase (IDO) promotes M2 macrophage polarisation | [87] |
|  | LINC00240 | Modulation of miR-155/Nrf2 axis promotes macrophage polarisation towards M2 type | [88] |
|  | LINC00221 | Negative regulation of miR-542-3p expression in trophoblasts reduces macrophage migration and invasion | [89] |
| 1. cell | miR-155 | Expansion of regulatory T cells enhances pregnancy tolerance | [109] |
|  | miR-33a/b  miR-181a | Negative regulation of S1PR1 expression leads to a decrease in the number of Treg cells | [110] |
|  | miR-106b | Negative regulation of Treg cell maturation with TNF-β | [111] |
|  | miR-20b | Positive regulation of transcription factors RORγt and STAT3 in TH17 cells | [111] |
|  | miR-363-3p | Increased TH17 activity | [111] |
|  | LncRNA Snhg7 | Regulation of CD4 T cell activation | [112] |
| Trophoblast | LncRNA DUXAP8 | Negative regulation of trophoblast function | [127] |
|  | Inc-HZ01 | Forms a positive feedback pathway with MXD1 and inhibits trophoblast cell proliferation | [128] |
|  | Circ_0003314 | Inhibition of trophoblast function and promotion of apoptosis by binding to miR-26b-5p and inducing high IL1RAP expression | [129] |
|  | Circ_0015382 | Targeted regulation of the miR-942-5p/NDRG1 axis impairs trophoblast function | [130] |
|  | Circ_0111277 | Regulation of miR-494-3p/HTRA1/Notch-1 signalling pathway inhibits trophoblast invasion and migration | [131] |

Table 2. Epigenetic regulation and placenta-associated pregnancy complications

| Epigenetic modifications | Molecules | Mechanisms | Diseases | Reference |
| --- | --- | --- | --- | --- |
| DNA Methylation | DNMT3A Downgrade | Mediates the non-dependent induction of DNA methylation by TGFBR1 | Early onset severe pre-eclampsia | [138] |
|  | Placental growth factor and Fms-associated tyrosine kinase-1 hypomethylation | —— | Pre-eclampsia | [139] |
|  | DNA methylation at the CPG site | —— | Low birth weight | [140] |
|  | Placental DNA methylation sites associated with birth weight | —— | Perinatal cardiometabolic status of the mother, chronic disease in later life of the offspring | [140] |
|  | CD3 methylation in pregnancy | —— | Psychiatric symptoms such as depression and anxiety | [142] |
| Histone modification | Deletion of Men1, a member of the histone H3K4 methyltransferase complex | Disruption of terminal differentiation of stromal cells | Embryo resorption and pregnancy failure | [145] |
|  | H3K18la downgrade | Influence on endometrial tolerance | Abortion | [146] |
|  | Knockout of KAT8 | Adjustment of H4K16ac/CDX2 axis | Vulnerable to embryo implantation failure induced miscarriage | [148] |
|  | KDM5CK upward | Regulate the expression of TGFβ2 and RAGE | Recurrent spontaneous abortion | [149] |
|  | H3K4me3 and H3K9ac downgrades | Regulated by Gal-2 and PPARγ | Pre-eclampsia | [150]  [151] |
|  | Down-regulation of HDAC 2 expression and activity in monocytes/macrophages | —— | Gestational diabetes | [153] |
|  | Up-regulation of miR-153-3p | Mediated inhibition of trophoblast function by the IDO/STAT3 pathway | Unexplained recurrent miscarriage | [152] |
| Non-coding RNA | Up-regulation of miR-185-5p | Reduced VEGF Expression and Angiogenesis in dNK Cells | Recurrent spontaneous abortion | [66] |
|  | Up-regulation of miR-515-5P | Reducing HDAC2 levels harms trophoblast cell biological behaviour | Recurrent spontaneous abortion | [153] |
|  | Up-regulation of miR-23a | Inhibition of HDAC2 and activation of NF-κB impede trophoblast migration and invasion and promote apoptosis | Pre-eclampsia | [154] |
|  | Down-regulation of miR-199a-5p | Reduction of VEGFA expression to inhibit trophoblast invasion | Pre-eclampsia | [155] |
| RNA methylation | Upregulation of METTL3 | Increase the level of m6A RNA methylation and hnRNPC1/C2 expression in trophoblasts | Pre-eclampsia | [157] |
|  | Upregulation of METTL14 | Increase the level of m6A RNA methylation and FOXO3a expression and inhibite trophoblast proliferation and invasion | Pre-eclampsia | [159] |
|  | Upregulation of RBM15 | RBM15 suppresses hepatic insulin sensitivity of offspring of gestational diabetes mellitus mice via m6A-mediated regulation of CLDN4 | Gestational diabetes Mellitus | [160] |
|  | Down-regulation of m6A methylation level | ALKBH5 regulates CYR61 mRNA stability through m6A dependent mechanism and affects trophoblast function | Recurrent spontaneous abortion | [132] |
|  | Down-regulation of METTL3 | METTL3 mediated ZBTB4 m6A RNA methylation modification inhibits trophoblast invasion ability | Recurrent spontaneous abortion | [161] |
